# Mental health literacy and its relationship with suicidal tendencies mediated by social support in students: a school-based study

**DOI:** 10.1186/s40359-025-03502-5

**Published:** 2025-11-03

**Authors:** Zeinab Habibpour, Mehrdad Karimi, Mohammad Saadati, Roghieh Sodeify, Morad Ali Zareipour

**Affiliations:** 1 Department of Nursing, Khoy University of Medical Sciences, Khoy, Iran; 2 Department of Public Health, Khoy University of Medical Sciences, Khoy, Iran

**Keywords:** Mental health literacy, Suicidal tendencies, Social support, Students, School, Adolescent

## Abstract

**Objective:**

Mental health challenges among adolescents have become a critical public health concern, particularly given the increasing prevalence of suicidal behaviours in this age group. While the relationships between mental health literacy, social support, and suicide risk have been studied globally, there is limited evidence from culturally specific contexts, particularly in less urbanized regions of Iran such as Khoy city. This study aimed to investigate mental health literacy) ability to understand and manage their own and others’ mental health (and its relationship with suicidal tendencies, mediated by social support, among students.

**Methods:**

This was a cross-sectional study conducted in 2024 in Khoy city, West-Azerbaijan, Iran. The study included 605 high-school students from six selected schools, who were selected through two-step random sampling.Mental Health Literacy, social support and suicide tendency questionnaires along with demographic information were used for data gathering. Data were analyzed using IBM SPSS AMOS Statistics 24 software. A structural equation modeling was employed to evaluate the relationships between the study variables and to test the conceptual framework.

**Results:**

The structural model results revealed significant direct and indirect associations among the variables. Mental Health Literacy (MHL) positively influenced the social support (β = 0.135, *P* = 0.03), while socioeconomic status (SES) also had a positive effect (β = 0.203, *P* < 0.001). Social support was found to negatively affect suicide tendency (β = -0.823, *P* < 0.001). Although MHL did not directly impact suicide tendency, it had a significant negative indirect effect through social support (β = -0.112, *P* = 0.04). SES also indirectly reduced suicide tendency (β = -0.167, *P* < 0.001). Living with parents had a direct negative effect on suicide tendency (β = -0.081, *P* = 0.04).

**Conclusions:**

Based on the findings of this study, the importance of integrating mental health literacy into curricula, establishing peer support networks, and strengthening family-school collaboration within existing frameworks such as the WHO Action Plan is emphasized. Furthermore, these results may provide a foundation for developing localized guidelines aimed at enhancing mental health literacy and social support in schools; however, longitudinal and intervention studies are necessary to confirm the effectiveness of these recommendations.

## Introduction

Mental disorders as a serious challenge of youth health affect approximately 2–20% of children and teens, globally. Despite this widespread prevalence, only one in five adolescents requiring mental health services actually receives care [1]. Mental health disorders are a well-known risk factor for increased suicide risk [2]. Nearly half of young people who die by suicide are reported to have had a mental health problem or diagnosis [3, 4]. Suicide is the tenth leading cause of death across all age groups and the second leading cause of mortality among youth [5]. The trend of suicide cases in some provinces of Iran shows that since 1990, the number of suicides among adolescents has been increasing [6].The assessment of suicidal thoughts among adolescents in schools in western Tehran in 1401 showed that the average score of suicidal thoughts among adolescents was at an average level (5.66 ± 3.19 out of 5) [7].Suicidal ideation among teenagers has emerged as a global public health concern resulting in high prevalence of youth suicide attempts [8, 9].

. In comparison to global rates, Iran is ranked among countries with a low prevalence of suicide attempts and completed suicides. However, while the rate of completed suicides has been declining in all ages, both suicide attempts and completed suicides have increased among younger age groups in Iran [10]. Suicide not only remains one of the leading causes of death in late childhood and teenagers globally but also leads to profound psychosocial and socioeconomic repercussions [5].

However, a major challenge in suicide prevention is that people often do not seek help when they are experiencing mental health problems [11] and often wait until they are in extreme distress [12]Therefore, as awareness of where to look for information about mental health increases, the willingness to communicate about suicide also increases significantly [13].

Mental health literacy is essential for adolescents’ well-being. Studies show that teenagers have low levels of mental health awareness and struggle to identify mental health issues and their related factors [14, 15]. A lack of mental health literacy is frequently cited as a significant barrier to seeking effective help among adolescents [16]. In a large-scale survey conducted among individuals under the age of 25 in the United Kingdom, half of the participants reported not seeking help for mental health problems due to a lack of understanding of their condition [17]. Mental health literacy involves understanding mental health and how to maintain it. Improving it can promote help-seeking behaviour and positive mental health outcomes [18]. It includes the ability to recognize mental health problems, build resilience, and engage in appropriate help-seeking behaviours [19, 20]. Mental health literacy provides a framework for mental health promotion, prevention, and care by integrating components that focus on improving mental health outcomes rather than just enhancing well-being [21]. Numerous studies have demonstrated that adequate mental health literacy is a crucial determinant of mental health promotion, particularly among individuals experiencing severe depression—a condition frequently associated with suicide-related outcomes [22, 23].

Social support also plays a pivotal role in improving mental health during the stressful period of being a teenager. Developing social connections and reducing concerns through support systems are major strategies to enhance mental health [24]. Studies have revealed a general effect of social support on predicting and influencing suicidal behaviour. Social support is defined as forces or factors in the environment that facilitate human survival [25]. It significantly delays suicidal ideation, and teenagers and young adults with limited social or familial support are more likely to exhibit self-destructive behaviors. Increased social support is associated with reduced exposure to suicidal thoughts among teen [24]. Furthermore, studies have reported low levels of perceived social support among individuals who attempt suicide, underscoring its importance as a critical factor in relation to suicidal ideation [26, 27].

While prior research has independently established the protective roles of mental health literacy (MHL) and perceived social support against adolescent suicidal tendencies, a significant theoretical gap remains in understanding the complex interplay and potential mechanisms linking these three constructs, particularly within the Iranian context. Specifically, there is limited evidence examining whether perceived social support mediates (e.g., MHL enhances perceived social support, which subsequently reduces suicidal tendencies) the relationship between MHL and suicidal tendencies among Iranian adolescents. Furthermore, existing studies in Iran often utilize broad or single-source measures of social support, potentially overlooking the multifaceted nature of support relevant to teenagers. This study aims to address this gap by concurrently investigating MHL, multidimensional perceived social support, and suicidal tendencies among high school students in Khoy, and by exploring the potential mediating pathway through which perceived social support influences the relationship between MHL and suicidal tendencies.

## Methods

### Design and setting

This was a cross-sectional study conducted in Khoy city high schools from October to November 2024. Khoy is the second most populated city in West-Azerbaijan province in the Northwest of Iran. The city has 9 health-promoting schools, covering 11,801 students (5977 girls, 5824 boys). Six of the nine health-promoting schools were high schools that were selected for the study. A Health Promoting School (HPS) constitutes a systemic framework for health enhancement that, through the active participation of parents, educators, and students and by adopting an empowerment approach towards students in the areas of self-care, fostering a culture of self-care, and peer education aims to build capacities and empower students for a healthy life.

### Study population and sample size

The study population consisted of all high-school students in Khoy city, Iran. The sample size was calculated using the formula for estimating a population mean. The following parameters were used: a Type I error (α) of 0.05 (Z = 1.96), and the standard deviation (σ) of the main variable, the total health literacy score. The mean and standard deviation were obtained from a pilot study on 26 randomly selected students from 3 different schools and were found to be 70.22 and 27.85, respectively. The desired margin of error (d) for the mean was set at 3.51 (5% of the pilot study mean).

The initial sample size was calculated as follows:

$$\:n=\frac{{Z}_{1-\alpha\:}^{2}\times\:{\sigma\:}^{2}}{{d}^{2}}=\frac{{1.96}^{2}\times\:{27.85}^{2}}{{3.51}^{2}}=242$$

Given that a multi-stage cluster sampling design was employed, a Design Effect (DEFF) was applied to adjust for the homogeneity of students within clusters (schools and classes). A conservative DEFF of 2.5 was chosen based on recommendations from WHO methodological guidelines for cluster surveys in educational settings where the intra-cluster correlation is unknown but anticipated to be moderate. Thus, the final required sample size was:

$$\:{n}^{{\prime\:}}=242\times\:DE=242\times\:2.5=605$$

### Sampling

A two-stage cluster sampling method was used. First, from the list of all nine health-promoting schools in Khoy (stratified by gender into 5 girls’ schools and 4 boys’ schools), three girls’ schools and three boys’ schools were selected using stratified random sampling. Within each gender stratum, schools were selected by simple random sampling using a random number generator. For the Class and Student Selection, in each selected school, all three grades (10, 11, and 12) were included. In each grade, which consisted of two classes, one class was selected per grade using systematic random sampling. This was done by listing the two classes in a random order, generating a random number (either 1 or 2), and selecting the corresponding class.

All students in the selected classes were invited to participate. With approximately 30–35 students in each class, we aimed to recruit about 100 students from each school. The final sample allocation was proportional to the gender distribution in the student population (51% girls). Therefore, 309 participants were allocated to girls’ schools and 296 to boys’ schools.

### Inclusion and exclusion criteria

The inclusion criteria for the study were as follows: students had to be enrolled in the upper secondary education level, be residents of urban areas, have no previously diagnosed psychological disorders, and provide informed consent to participate in the research. On the other hand, exclusion criteria were also applied, according to which participants who had incompletely filled out the questionnaires or had not provided parental consent for participation in the study were excluded. In total, 605 eligible students were initially enrolled in the study. Of these, based on the exclusion criteria, 41 individuals (7.7%) were excluded. The reason for exclusion for 30 cases (72.9% of excluded cases) was failure to provide parental consent, and for the remaining 11 cases (26.8%), it was incomplete filling of the questionnaires. Ultimately, data from 564 individuals (93.2% of the initial sample) were included in the final analysis.

### Data gathering

Three main tools as following were used for data gathering alongside with demographic information.

### Demographic information

The demographic questionnaire captured data on the student’s age, gender, and grade level, alongside parental educational attainment and occupational status. Furthermore, the family’s economic status was assessed through a self-rated measure, evaluated relative to their peers and categorized as ‘good,’ ‘middle, ‘poor.’ The household living arrangement, specifying whether the student resided with parents or other guardians, was also documented. Finally, parental age was included in this questionnaire.

### Mental health literacy

The tool originally was developed by Zarebi et al. [1] for teen and teenager audiences. It includes 29 items in 4 dimensions as Knowledge of Mental Health Problems, Erroneous Beliefs and Stereotypes, First aid skills and help-seeking behaviours and self-help strategies. The answers are in 5-point Likert scale from completely agree to completely disagree. The scale score range is from 29 to 145 and higher score shows higher mental health literacy. The tool validation is reported in previous published literature and the α = 0.75 [1].

### Social support

This was originally developed in 1974 by Philips et al. and includes 23 questions in family, friends and others dimensions. Each question is answered true or false allocating 0 and 1, respectively. The score is ranged from 0 to 29 as the higher score is a sing of higher social support. The Persian version of the tool was validated in Iran by Nasseh et al. with α = 0.67 [28].

### Suicide tendency

This multifaceted tool was developed by Urbakh et al. in 1981 which encompass 30 items in 4 dimensions of attraction to life, repulsion by Life, attraction to death and repulsion by death. The answers for each item is in a 5-point Likert scale as ‘highly disagree’, ‘disagree’, ‘sometimes agree, sometimes disagree’, ‘agree’, ‘highly agree’. The Persian version of the tool was validated by Sheikh et al. [29]

### Data collection procedure

Upon obtaining the necessary permits, the research team visited the selected schools in person to conduct operational and executive coordination. During informative sessions held with school administrators, the study’s objectives, ethical protocol, and detailed procedures were transparently explained. Furthermore, a precise and timed plan for data collection was established, taking into account the need to avoid disruption to the students’ educational process. Then, based on the plan, they attend in the classes with the class teacher. After explaining the study goals to the students by researchers, they were requested to fill the questionnaire. Filling the questionnaire took approximately 15 min. Data collection was conducted over a two-month period, extending from the beginning of October to the end of November 2024.

### Data analysis

The mean and standard deviation of quantitative data and the frequency (percentage) of qualitative data are reported as descriptive statistics. Pearson correlation test used to assess the association among study variables. In this study, structural equation modeling (SEM) was used to test the relationships between variables. The software AMOS 24 was utilized for this purpose. The parameter estimation method employed was Maximum Likelihood Estimation (MLE). Appropriate fit indices, such as the Chi-square test (χ²), Root Mean Square Error of Approximation (RMSEA), Comparative Fit Index (CFI), Goodness of Fit Index (GFI), Adjusted Goodness of Fit Index (AGFI), Normed Fit Index (NFI), Incremental Fit Index (IFI), and Standardized Root Mean Square Residual (SRMR), were used to evaluate the model’s fit and suitability. Structural Equation Modeling (SEM) is a comprehensive statistical approach used to test hypotheses about relationships among observed and latent variables. It combines elements of factor analysis and multiple regression analysis, allowing researchers to examine complex relationships in a single model. The key components of SEM include latent variables, which are unobserved variables inferred from observed variables (indicators), and observed variables, which are directly measured variables. The measurement model specifies how latent variables are measured by observed variables, while the structural model specifies the relationships among latent variables. SEM offers several advantages, including the ability to perform simultaneous analysis of multiple relationships, account for measurement error in the estimation process, and handle complex models with multiple dependent and independent variables. By using SEM, researchers can test theoretical models and assess the fit of the model to the data using various fit indices [30, 31].

### Ethics approval

The university’s ethics committee emphasized the importance of informed consent from students and parents. During the data collection process involving students, written informed consent was obtained from both the students and their parents.

## Results

### Study population

Table 1 presents demographic details of 564 students ranged between 16 and 19 years old. The mean age of the students was 17.52 ± 0.94 years. Most of the students were male (52.5%), the economic status of the families was reported as 7.6% weak, 56.4% middle, and 36.0% good. In terms of mothers’ education levels, 4.3% were uneducated and 16.0% had a university degree. For fathers’ education levels, 1.2% were uneducated and 21.5% had a university degree. Regarding occupation, 91.5% of the mothers were household workers, while 8.5% were employed. Among fathers, 76.8% were Unemployed, and 23.2% were employed. Most participants (94.1%) lived with their parents, while 5.9% lived with others. The mean age of the mothers and fathers were 42.49 ± 5.78 and 48.16 ± 6.05 year, respectively.

**Table 1:** The characteristics of study population

Variables	*n* (%), mean ± SD
Age	17.52 ± 0.94
Gender	
male	296 (52.5)
female	268 (47.5)
Grade	
1	184 (32.6)
2	193 (34.2)
3	187 (33.2)
Mother’s education	
uneducated	24 (4.3)
primary	107 (19.0)
high school	113 (20.0)
diploma	230 (40.8)
university	90 (16.0)
Father’s education	
uneducated	7 (1.2)
primary	108 (19.1)
high school	133 (23.6)
diploma	195 (34.6)
university	121 (21.5)
Mother’s occupation	
household	516 (91.5)
employed	48 (8.5)
Father’s occupation	
Unemployed	433 (76.8)
employed	131 (23.2)
Economic status	
weak	43 (7.6)
middle	318 (56.4)
good	203 (36.0)
Live with*	
parents	531 (94.1)
other	33 (5.9)
Mother’s age	42.49 ± 5.78
Father’s age	48.16 ± 6.05

* Living with parents refers to living with both father and mother in the same household. Living with others refers to living with only the father, only the mother, or with other guardians

### Main study variables and their reliability

Table 2 presents the mean and standard deviation of the main variables and their subscales, along with reliability indices including Cronbach’s alpha and composite reliability (CR). Cronbach’s alpha measures internal consistency, with values of 0.70 or higher considered acceptable. Composite reliability (CR) is a more conservative measure of internal consistency, also considered acceptable at 0.70 or higher. In this study, all scales demonstrated acceptable levels of internal consistency, with Cronbach’s alpha and CR values close to or exceeding 0.70, confirming the reliability of the measurement scales.

**Table 2:** The mean and standard deviation of the study variables, along with their reliability evaluations

Variables	No of items	Mean	SD	Cronbach’s alpha	CR*
Mental Health Literacy	29	109.30	11.57	0.78	0.83
Knowledge of Mental Health Problems	11	41.18	5.28	0.72	0.81
Erroneous Beliefs and Stereotypes	8	29.82	4.78	0.67	0.78
First-Aid Skills and Help-Seeking Behavior	6	22.44	4.39	0.70	0.81
Self-Help Strategies	4	16.66	2.37	0.68	0.80
Social Support	23	17.05	3.80	0.75	0.83
family	8	6.51	1.60	0.73	0.82
friends	7	4.99	1.94	0.74	0.82
other	8	5.54	1.71	0.70	0.78
Suicide Tendency		77.44	12.3	0.75	0.84
Attraction to Life	7	25.51	5.44	0.76	0.84
Repulsion by Life	7	16.92	5.28	0.74	0.81
Attraction to Death	7	15.85	5.53	0.75	0.86
Repulsion by Death	9	19.17	7.55	0.88	0.90

* Composite reliability

### Pearson correlation matrix of the study variables

Table 3 presents the Pearson correlation matrix for the study variables, showing the correlation coefficients and *p*-values for each pair of variables. The analysis indicates that mental health literacy is positively and significantly correlated with social support (*r* = 0.21, *P* < 0.05). Specifically, the dimensions of “first-aid skills and help-seeking behavior” (*r* = 0.37, *P* < 0.01) and “self-help strategies” (*r* = 0.09, *P* < 0.05) within mental health literacy also show positive and significant correlations with social support. Additionally, mental health literacy is negatively and significantly associated with the suicide tendency(*r* = −0.11, *P* < 0.05). Among the dimensions of mental health literacy, “first-aid skills and help-seeking behavior” (*r* = −0.16, *P* < 0.01) has a negative and significant relationship with the suicide tendency. Social support itself is negatively and significantly related to the suicide tendency(*r* = −0.15, *P* < 0.01). Furthermore, family support (*r* = −0.23, *P* < 0.01) and support from others (*r* = −0.09, *P* < 0.05) also exhibit negative and significant relationships with the suicide tendency.

**Table 3:** Pearson correlation test among mental health literacy, social support, and suicide tendency in students

Variables	1	2	3	4	5	6	7	8	9	10	11	12	13
1. Mental Health Literacy													
2. Knowledge of Mental Health Problems	0.81^**^												
3. Erroneous Beliefs and Stereotypes	0.69^**^	0.43^**^											
4. First-Aid Skills and Help-Seeking Behavior	0.55^**^	0.25^**^	0.05										
5. Self-Help Strategies	0.69^**^	0.56^**^	0.35^**^	0.26^**^									
6. Social Support	0.21^**^	0.07	0.05	0.37^**^	0.09^*^								
7. Family	0.17^**^	0.06	0.06	0.28^**^	0.08^*^	0.64^**^							
8. Friends	0.11^**^	0.02	0.02	0.21^**^	0.05	0.74^**^	0.13^**^						
9. Others	0.18^**^	0.09^*^	0.03	0.32^**^	0.06	0.77^**^	0.33^**^	0.39^**^					
10. Suicide Tendency	−0.11^**^	−0.06	−0.06	−0.16^**^	−0.02	−0.15^**^	−0.23^**^	−0.02	−0.09^*^				
11. Attraction to Life	0.29^**^	0.12^**^	0.23^**^	0.24^**^	0.20^**^	0.39^**^	0.33^**^	0.13^**^	0.30^**^	0.13^**^			
12. Repulsion by Life	−0.22^**^	−0.09^*^	−0.08	−0.34^**^	−0.10^*^	−0.43^**^	−0.48^**^	−0.14^**^	−0.34^**^	0.63^**^	−0.37^**^		
13. Attraction to Death	−0.10^*^	−0.02	0.01	−0.26^**^	−0.02	−0.27^**^	−0.29^**^	−0.07	−0.24^**^	0.59^**^	−0.28^**^	0.60^**^	
14. Repulsion by Death	−0.16^**^	−0.10^*^	−0.23^**^	0.01	−0.09^*^	0.01	−0.06	0.02	0.06	0.67^**^	−0.05	0.16^**^	0.01

* *P* < 0.05

** *P* < 0.01

### Structural models

#### Initial model

**Fig. 1:**
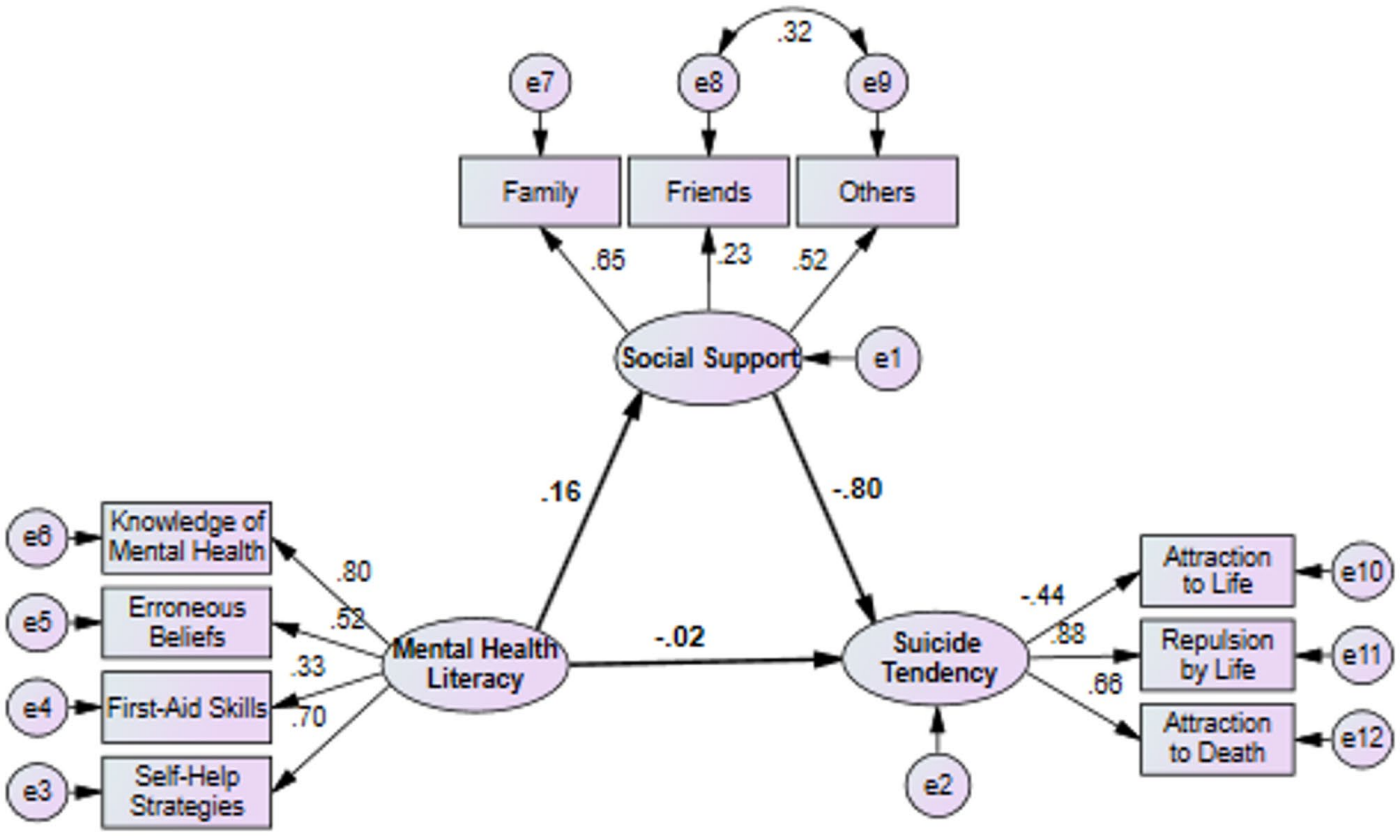
Modified model of the interrelationship among mental health literacy, social support, and suicide tendency in students. Fit indices: Chi-square = 89.02, DF = 29, Chi-square to DF = 3.07, SRMR = 0.049, RMSEA = 0.061, GFI = 0.97, AGFI = 0.94, NFI = 0.93, IFI = 0.95, CFI = 0.91, TLI = 0.92

The initial structural model analysis revealed a significant positive direct effect of mental health literacy on social support (β = 0.157, *P* = 0.02). However, its direct effect on suicide tendency was not significant (β = −0.016, *P* = 0.73). Social support demonstrated a significant negative direct effect on suicide tendency (β = −0.80, *P* < 0.001). Crucially, a significant negative indirect effect of mental health literacy on suicide tendency was observed, mediated entirely by social support (β = −0.126, *P* = 0.02), confirming the mediating role of social support in the model. All indicator loadings for the constructs were significant (*P* < 0.001) except for one indicator (‘repulsion by death’) in the suicide tendency construct, which was subsequently removed. The model’s fit indices, as shown in Fig. 1, confirmed an acceptable fit to the data.

#### Adjusted structural model and modification process

**Fig. 2:**
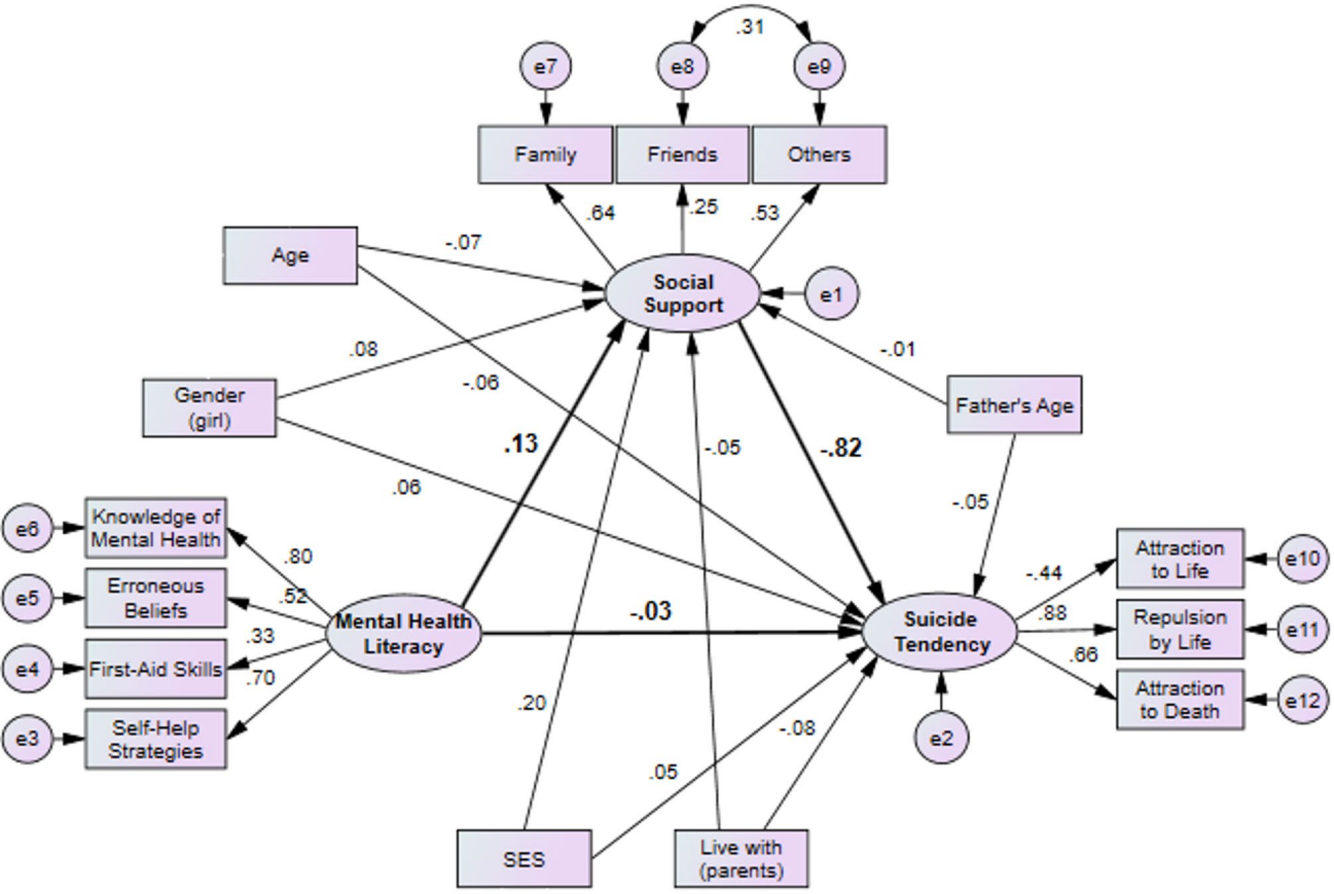
Modified adjusted model of the interrelationship among mental health literacy, social support, and suicide tendency in students. Fit indices: Chi-square = 189.17, DF = 76, Chi-square to DF = 2.49, SRMR = 0.052, RMSEA = 0.051, GFI = 0.96, AGFI = 0.93, NFI = 0.88, IFI = 0.92, CFI = 0.92, TLI = 0.89

The development of the final structural model was conducted through a systematic process to ensure both statistical adequacy and theoretical relevance. Initially, a comprehensive model was tested, which included all predefined pathways between the latent constructs and all collected demographic variables as covariates—namely, age, gender, father’s age, socioeconomic status (SES), living with parents, and parents’ education and occupation. This initial, saturated model, however, did not demonstrate an acceptable fit to the data, as indicated by the following fit indices: χ²/df = 3.428, SRMR = 0.078, RMSEA = 0.066, CFI = 0.831, TLI = 0.781. To refine the model, a theory-guided reduction approach was undertaken. Covariates that did not contribute significantly to the model (with *p*-values greater than 0.10) were sequentially considered for removal. The indicator ‘Repulsion by Death’ was removed from the measurement model prior to this analysis due to a non-significant factor loading (*p* > 0.05), a standard practice to ensure measurement validity. Consequently, the variables of parents’ education and occupation were excluded from the subsequent analyses due to their statistical non-significance (*P* > 0.10). The final adjusted model retained the covariates of age, gender, father’s age, SES, and living with parents, which were deemed to have stronger theoretical justification for their potential influence on the core constructs. This final model demonstrated a significantly improved and excellent fit to the data (χ² = 189.17, df = 76, χ²/df = 2.49, RMSEA = 0.051, SRMR = 0.052, CFI = 0.92, TLI = 0.89, GFI = 0.96), as presented in Fig. 2. The results from this parsimonious final model, detailed in Fig. 2 and Table 4, revealed a series of significant direct and indirect associations:

**Table 4:** Structural model results: standardized direct and indirect associations among variables based on modified model

		Direct effect		Indirect effect	
Independent	Dependent	β	*P*-value	β	*P*-value
MHL	Social support	0.135	0.03		
age		−0.068	0.22		
gender (girl)		0.083	0.12		
father’s age		−0.007	0.89		
SES		0.203	< 0.001		
live with (parents)		−0.051	0.34		
Social support	Suicide tendency	−0.823	< 0.001		
MHL		−0.027	0.62	−0.112	0.04
age		−0.059	0.22	0.056	0.25
gender (girl)		0.063	0.17	−0.069	0.16
father’s age		−0.046	0.32	0.006	0.88
SES		0.047	0.34	−0.167	< 0.001
live with (parents)		−0.081	0.04	0.042	0.37

*MHL* Mental Health Literacy

Mental Health Literacy (MHL) positively affects social support (β = 0.135, *P* = 0.03), and socioeconomic status (SES) also positively influences social support (β = 0.203, *P* < 0.001). However, age, gender, father’s age, and living with parents do not have significant direct effects on social support (*P* > 0.05). Social support negatively affects suicide tendency (β = −0.823, *P* < 0.001), indicating higher social support is linked to lower suicide tendency. MHL does not directly affect suicide tendency (β = −0.027, *P* = 0.62) but has a significant negative indirect effect through social support (β = −0.112, *P* = 0.04). Age, gender, and father’s age do not have significant direct or indirect effects on suicide tendency (*P* > 0.05). SES has a significant negative indirect effect on suicide tendency (β = −0.167, *P* < 0.001), suggesting higher SES reduces suicide tendency through increased social support. Living with parents has a significant negative direct effect on suicide tendency (β = −0.081, *P* = 0.04), but its indirect effect is not significant (*P* > 0.05). (Table 4).

These results highlight the importance of social support and socioeconomic status in influencing mental health outcomes, particularly in reducing suicide tendency. Mental health literacy also plays a crucial role indirectly through its impact on social support. Specifically, social support acts as a mediator in the relationship between mental health literacy and suicide tendency, as well as between socioeconomic status and suicide tendency. This mediation effect underscores the critical role of social support in mitigating the risk of suicide by enhancing the positive effects of mental health literacy and socioeconomic status.

## Discussion

According to the results, mental health literacy could lower the student’s Suicidal tendency social support promotion, but it did not have a direct effect on students’ s Suicidal tendency. However, social support plays a significant role as it has a direct relationship with the reduction of Suicidal tendency and acts as an important mediator between mental health literacy and Suicidal tendency. Yang et al. demonstrated that mental health literacy has a positive effect on perceived social support and increases the likelihood of individuals seeking professional psychological help [32]. Specifically, Mental health literacy improvement among stuents could lead to reduced stigma and creates a supportive environment encouraging help-seeking behaviors among them. On the other hand, Bennett et al. demonstrated that a sufficient level of mental health literacy significantly improves individuals’ capacity to identify their mental health status and provide peer support, which in turn improves social support networks among Australian youth and positively impacts mental health outcomes [33]. Similarly, Jung et al. study results showed that mental health literacy positively affects attitudes towards mental health help-seeking behaviors, which may increase social support (30). Nevertheless, regarding the contradictory findings, the relationship between mental health literacy and social support requires further investigations [32].

Beyond regional evidence, According to the World Health Organization, over 720,000 people worldwide die by suicide annually, and 73% of these cases occur in low- and middle-income countries [34]. For instance, a study from Southeast Asian (ASEAN) countries on students revealed a high prevalence of mental health problems (depression, anxiety, stress, suicide); however, the tendency to seek professional help is relatively low [35]. Conversely, Western findings also indicate the protective role of social support in reducing depression, anxiety, and suicidal thoughts/attempts; in a study by Scardera et al. in Canada, it was shown that individuals who perceived higher social support at age 19 experienced fewer depressive symptoms and suicidal thoughts the following year [36].

Collectively, these international evidences indicate that the role of mental health literacy and social support as protective factors against Suicidal tendency is consistent worldwide, and our study’s findings are aligned with these patterns.

Regarding the association between social support and suicidal tendency, Dennehy et al. study revealed that social support from parents, classmates, instructors, and close friends serves as a protective mediator. Support from close friends was found to be a stronger buffer for girls [37]. In a study in Iran, Arab et al. similarly demonstrated a significant negative correlation between perceived social support and suicidal thoughts, with support from friends and family being the most substantial predictor of suicidal ideation [38]. These results collectively imply that improving mental health literacy should be accompanied by strengthening social support networks. Therefore, educational and supportive programs including targeted social support and community-based interventions should be in place not only to increase mental health literacy but also to create and strengthen social support networks.

When it came to the sources of social support, family support was shown to be the most significant determinant of social support among students. Daly’s research indicated that family support is a major component of social support among students. Family support includes various forms such as instrumental, informational, and emotional support, all of which help in students adjustment to the school and college life [39]. Furthermore, the results showed that students living with their parents had a significant reduced suicidal ideation. According to Flouri’s study, that teenagers who live with both parents report less suicidal thoughts than those living alone or with people other than their parents [40]. Additionally, Minullina reported that adolescents who have harmonious relationships with their parents are less likely to have suicidal tendencies, as discordant relationships, especially with mothers, are associated with higher risks [41].Thus, Strengthening family-based social support can help students feel less hopeless and depressed which indirectly decrease suicidal ideation. As a result, promoting family engagement in mental health initiatives in schools, developing family-school collaboration models through enhancing communication channels and considering interventions for at-risk students with their family participation could help to promote familiar support network.

Regarding the components of mental health literacy, the results indicated that the most significant determinants of mental health literacy were the dimensions of Knowledge of Mental Health Problems and Self-Help Strategies. According to Soria et al. mental health literacy is greatly influenced by awareness of mental health problems and self-help strategies [42]. Venkataraman et al. found that improving awareness about mental health issues and self-help techniques can positively affect mental health literacy. This underscores the importance of raising public knowledge on self-identification to improve help-seeking behaviors and treatment outcomes [39]. These findings suggest that to improve mental health literacy among students, there should be a focus on promoting their knowledge and awareness regarding mental health problems as well as self-help and self-care strategies. Incorporating evidence-based mental health education in the curriculums could provide an opportunity for students to be more knowledgeable about mental health [43].

Regarding the dimensions of Suicidal tendency, Repulsion by Life and Attraction to Death were the most significant determinants of suicidal ideation among students. Orbach’s study showed that increased Repulsion by Life and Attraction to Death lead to greater suicidal ideation, and these two dimensions are among the most important factors determining suicidal ideation in students [44]. Moreover, Mohammadi et *al.* [45] linked the higher levels of depression to Attraction to Death dimension. Students who showed a greater attraction to death often report less attraction to life, indicating a propensity for Suicidal tendency. Mental health screening programs, targeted psychological interventions, family and teacher’s education and counselling and peer support networks could enhance the schools capacity in identifying the early warning signs of reduced attraction of life, enabling timely support for at-risk students.

Through greater social support, higher economic standing students were less likely to have Suicidal tendency. Näher et al. reported that higher economic status was associated with lower suicide rates [46]. An improved socio-economic status fosters more social support, which reduce the negative effects of social isolation on mental health [47]. Gültekin et al. [48] The study results underlined an adverse correlation between suicide rate and average per capita income at the macro-socioeconomic level. They highlighted that improving economic standing may potentially reduce suicidal ideation by providing more opportunities for community support and education. At the family level, when families are in a better economic position, they are generally able to provide more resources to meet their children’s emotional and social needs. Consequently, at the individual level, students experience greater psychological benefits as a result of feeling more supported and secure. Additionally, access to social and recreational activities, often associated with higher economic status, can improve interpersonal links, social relationships, and create effective support networks. These findings emphasize the need for multi-level interventions: Targeted interventions for economically disadvantaged students (individual level) and collaboration for economic empowerment of families ༈family level༉ would potentially play a protective role in this regard.

### Limitations and suggestions for future research

Despite the valuable findings, this study had several limitations. The cross-sectional design limits the ability to infer causal relationships between mental health literacy, social support, and suicidal tendencies. Furthermore, the generalizability of the findings requires caution due to the sampling of students from a single city. Additionally, all data were self-reported, which increases the risk of social desirability bias, particularly for sensitive questions such as those related to suicidal tendencies.

Based on these limitations, prospective longitudinal studies are recommended to examine causal relationships among the variables over time. Expanding sampling to more diverse geographical and cultural regions is also essential to enhance the generalizability of the results. Employing qualitative approaches, such as interviews, in future studies could provide deeper insights into the underlying mechanisms of the phenomenon under investigation.

## Conclusion

The findings of this present cross-sectional study demonstrate significant associations between mental health literacy, social support systems, and suicidal tendencies among student populations. The results indicate that mental health literacy appears to be indirectly associated with reduced suicidal tendency, potentially mediated through enhanced social support mechanisms. Within these support networks, familial support emerged as a particularly significant component, showing consistent associations with reduced levels of hopelessness and suicidal tendencies. Furthermore, higher socioeconomic status was correlated with lower suicide ideation, possibly attributable to increased access to psychological resources and social opportunities. The psychological constructs of repulsion by life and attraction to death were identified as significant factors associated with suicidal ideation, both of which frequently co-occur with depressive symptoms. It is crucial to emphasize that, Given the cross-sectional design of this study, the findings indicate associations without implying causality. The methodological approach limits the ability to draw definitive conclusions about the direction or cause-effect relationships between variables. Despite this limitation, the current results can inform the development and implementation of comprehensive school-based mental health initiatives at national and regional levels. Specifically, within frameworks such as the WHO Mental Health Action Plan and ASEAN School Mental Health Promotion programs, evidence-informed strategies could be considered: (1) integrating mental health literacy education into standard curricula, (2) establishing peer support networks, and (3) enhancing family-school collaboration frameworks [49]. These findings may also support the creation of localized guidelines for improving mental health literacy and strengthening social support systems in educational settings, consistent with WHO recommendations for community-based, multi-level suicide prevention interventions among adolescents. This approach may enhance individual awareness and coping skills while fostering supportive environments through the active involvement of families, educators, and peers. However, future longitudinal studies and intervention trials are necessary to verify causal relationships and assess the effectiveness of these preventive strategies.

## Data Availability

All data generated in this study are included in the manuscript. Datasets are available upon reasonable request from the corresponding author.

## Abbreviations

MHL: Mental health literacy
SS: Social support
ST: Suicide tendency
HPS: Health promoting school
SES: Socio-economic status
CR: Composite reliability
WHO: World Health Organization
ASEAN: Association of southeast Asian Nations
